# An ethogram analysis of cutaneous thermal pain sensitivity and oxycodone reward-related behaviors in rats

**DOI:** 10.1038/s41598-023-36729-6

**Published:** 2023-06-28

**Authors:** Ariana C. Brice-Tutt, Darrice S. Montgomery, Cassidy M. Kramer, Peter M. Novotny, Wendi L. Malphurs, Abhisheak Sharma, Robert. M. Caudle, Adriaan W. Bruijnzeel, Barry Setlow, John K. Neubert, Niall P. Murphy

**Affiliations:** 1 Departments of Orthodontics, University of Florida, Gainesville, FL USA; 2 Department of Pharmaceutics, University of Florida, Gainesville, FL USA; 3 Department of Oral and Maxillofacial Surgery, University of Florida, Gainesville, FL USA; 4 Department of Psychiatry, University of Florida, Gainesville, FL USA; 5 Center for Addiction Research and Education, University of Florida, Gainesville, FL USA

**Keywords:** Motivation, Reward, Neuropathic pain

## Abstract

Inter-relationships between pain sensitivity, drug reward, and drug misuse are of considerable interest given that many analgesics exhibit misuse potential. Here we studied rats as they underwent a series of pain- and reward-related tests: cutaneous thermal reflex pain, induction and extinction of conditioned place preference to oxycodone (0.56 mg/kg), and finally the impact of neuropathic pain on reflex pain and reinstatement of conditioned place preference. Oxycodone induced a significant conditioned place preference that extinguished throughout repeated testing. Correlations identified of particular interest included an association between reflex pain and oxycodone-induced behavioral sensitization, and between rates of behavioral sensitization and extinction of conditioned place preference. Multidimensional scaling analysis followed by k-clustering identified three clusters: (1) reflex pain, rate of behavioral sensitization and rate of extinction of conditioned place preference (2) basal locomotion, locomotor habituation, acute oxycodone-stimulated locomotion and rate of change in reflex pain during repeated testing, and (3) magnitude of conditioned place preference. Nerve constriction injury markedly enhanced reflex pain but did not reinstate conditioned place preference. These results suggest that high rates of behavioral sensitization predicts faster rates of extinction of oxycodone seeking/reward, and suggest that cutaneous thermal reflex pain may be predictive of both.

## Introduction

There is considerable interest in the fundamental mechanisms of pain and pleasure, especially the degree to which underlying neural systems overlap and mutually impact these two psychological domains^1–5^. Practically, much of this interest is driven by the goal of understanding how individual differences in pain sensitivity impact vulnerability to substance use disorders^6,7^. Evidence suggests the experience of pain is comorbid with various mood and anxiety disorders^8–10^, and of particular interest to the current study, individuals more sensitive to pain are suggested to be more likely to develop opioid use disorders^11–13^.

Despite significant concern over their abuse liability, opioids remain common for managing severe pain^14^. Among these, the synthetic opioid oxycodone (OXY) is particularly concerning as it ranks highly among misused substances^15–17^. Preclinical studies agree that OXY exhibits many of the characteristics of an addictive substance. For instance, animal studies show that OXY exposure induces conditioned place preference^18–23^, is readily intravenously self-administered^24–28^, induces behavioral sensitization^29^ and in some, but not all studies, OXY solutions are preferred over water via oral consumption^30–32^. Moreover, OXY exhibits the hallmark action of stimulating mesolimbic dopamine release^33^.

Generally, the relationship between individual differences in pain sensitivity and subsequent vulnerability to opioid misuse, especially OXY, has been little studied. Here we capitalized on the ability of the conditioned place preference (CPP) paradigm to provide multiple measures related to abuse liability to assess relationships between thermal pain sensitivity in drug-naïve animals and behavioral measures relevant to vulnerability to OXY misuse. That is, in addition to assaying the rewarding properties of drugs, the CPP paradigm delivers useful information on the psychomotor activating and behavioral sensitizing effects of drugs of abuse. Additionally, through extended testing, the CPP paradigm can model extinction of drug-seeking behavior. Finally, the CPP paradigm offers insight into some of the processes that reinstate drug-seeking behavior, a.k.a. relapse. For instance, drug re-exposure and stress readily reinstate drug-seeking behaviors in CPP^34,35^. We performed an ethogramic analysis of relationships between reflex cutaneous thermal pain and OXY-related drug-seeking behavior. We found that locomotor and locomotor habituation measures were generally positively related, and that rates of behavioral sensitization and extinction of conditioned place preference associated with basal reflex pain measures. Contrary to expectation, however, induction of a chronic pain state through chronic nerve constriction (CCI) did not reinstate extinguished OXY-induced CPP.

## Methods

### Overview of study

**Figure 1:**
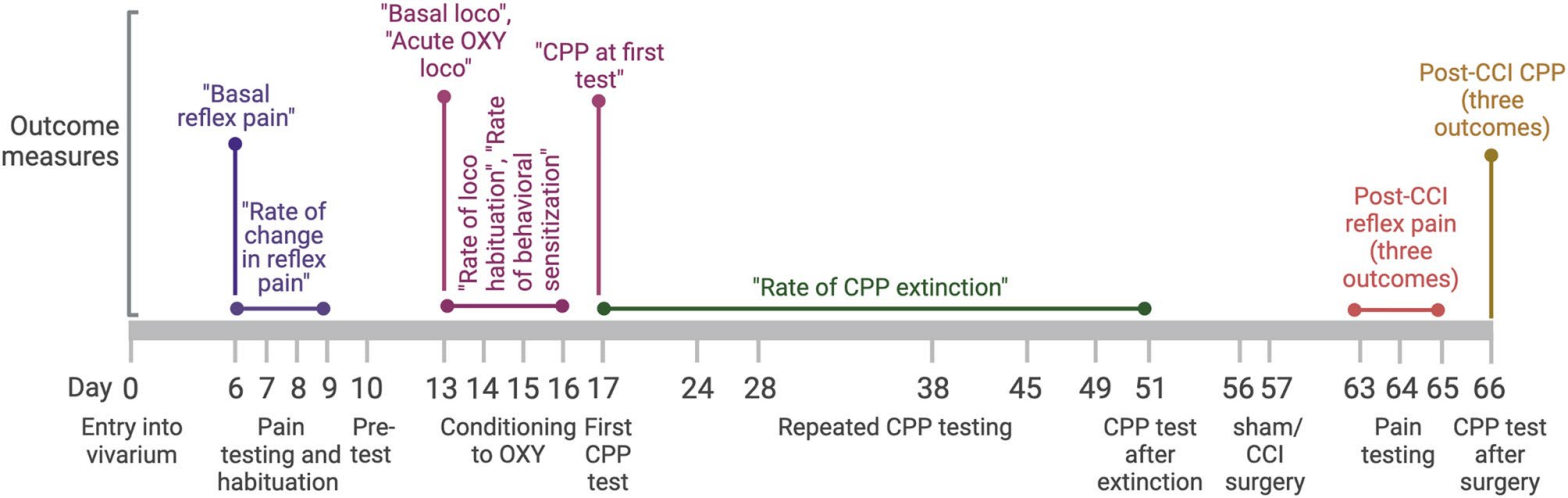
Schematic representation of experimental design and timings of behavioral outcomes.

The study was performed in five major phases, as depicted in Fig. 1. The first phase (pain testing) consisted of four sessions of measuring reflexive hindpaw withdrawal latencies from a cutaneous thermal nociceptive stimulus (referred to as "reflex pain') performed in single sessions over 4 days. Over the following 8 days (days 10 to 17) animals underwent the second phase – induction of CPP – consisting of a pre-test of initial compartment preference (day 10), 4 days of conditioning (days 13 to 16) in which each day, animals received both vehicle (VEH) and OXY conditioning sessions, and a test of CPP (day 17). Following this, in phase 3, animals underwent extinction testing in which they were re-tested for CPP expression 7 (day 24), 11 (day 28), 21 (day 38), 28 (day 45), 32 (day 49) and 34 (day 51) days after the initial test of CPP expression performed immediately following conditioning. In phase 4 (days 56 and 57), animals were pseudorandomly counterbalanced by sex and CPP score on day 51 into two groups that received either chronic constriction injury (CCI) of the sciatic nerve or a sham operation procedure. Following this, animals underwent three sessions of thermal hindpaw reflex pain testing performed in single sessions over days 63 to 65. In the final phase, the impact of CCI surgery on CPP expression was tested on day 66. Various outcomes were recorded at different stages of the study, which are detailed in Tables 1 and 2 and explained below. Studies are reported in accordance with ARRIVE guidelines. Before sham/CCI surgery all animals were exposed to the same experimental conditions, and thus there was no independent factor to mask. Following sham/CCI surgery, the investigator performing cutaneous thermal pain testing was the same investigator that performed sham/CCI surgery, though the investigator was not explicitly aware of the type of surgery that each animal had undergone. CPP measures made after sham/CCI surgery were made by investigators blind to the surgery status of the animals. Data were analyzed by an investigator that had no direct involvement in executing the studies, but who was aware of sham/CCI treatment allocation at the initiation of analysis.

**Table 1:** Summary of behavioral measures acquired before sham/CCI surgery.

Outcome	Measure	Unit	Shapiro–Wilk normality	Calculation method	Interpretation
Basal reflex pain	Reflexive paw withdrawal latency from a cutaneous thermal nociceptive stimulus	s	W = 0.897, p = 0.085	Paw withdrawal latency averaged across both paws in the first test (day 6) before induction of CPP	High values reflect lower cutaneous thermal pain sensitivity
Rate of change in reflex pain	Rate of habituation/sensitization to repeated cutaneous thermal nociceptive stimulation	s/session	W = 0.938, p = 0.355	Inverted slope of linear regression of average withdrawal latencies across both paws during testing (days 6 to 9) before induction of CPP	High values reflect increased sensitization of cutaneous thermal pain over test sessions
Basal loco	Locomotion	Counts	W = 0.966, p = 0.801	Absolute beam break counts following VEH administration in the VEH-paired compartment on the first day (day 13) of conditioning in the CPP apparatus	High values reflect high undrugged locomotion
Rate of loco habituation	Rate of locomotor habituation	Counts/session	Pre- transform: W = 0.866, p = 0.030	Square root transformed inverted slope of linear regression of beam break counts following VEH in the VEH-paired compartment during conditioning (days 13 to 16) in the CPP apparatus	High values reflect rapid locomotion habituation
			Post-transform: W = 0.940, p = 0.383		
Acute OXY loco	Locomotor response to OXY	Counts	W = 0.898, p = 0.090	Absolute beam break counts following OXY on the first day of conditioning (day 13) normalized by subtraction of absolute beam break counts following VEH on the same day in the CPP apparatus	High values reflect high psychomotor response to OXY
Rate of behavioral sensitization	Rate of behavioral sensitization to OXY	Counts/session	W = 0.925, p = 0.231	Slope of linear regression of VEH-normalized locomotion following OXY over the four conditioning sessions (day 13 to 16) in the CPP apparatus	High values reflect rapid behavioral sensitization
CPP at first test	CPP to the OXY-paired compartment immediately following conditioning	s	W = 0.943, p = 0.426	Subtraction of time in the OXY-paired compartment before conditioning (Pre-test, day 10) from time in the OXY-paired compartment immediately after conditioning (day 17)	High values reflect stronger CPP
Rate of CPP extinction	Rate of extinction of CPP following conditioning	s/day	W = 0.958, p = 0.651	Inverted slope of linear regression of CPP scores calculated the same as "CPP at first test" during repeated testing (days 17 to 51) following conditioning	High values reflect rapid extinction of CPP

**Table 2:** Summary of behavioral measures acquired after sham/CCI surgery.

Outcome	Measure	Unit	Shapiro–Wilk normality	Calculation method	Interpretation
Reflex pain–surg. paw	Paw withdrawal latency from cutaneous thermal stimulation in the surgeried paw following sham/CCI surgery	s	W = 0.929, p = 0.328	Average paw withdrawal latency of the surgeried paw across the three test sessions (days 63 to 65) following sham/CCI surgery	High values reflect lower cutaneous thermal pain sensitivity
Reflex pain–surg. paw vs. non-surg. paw	Relative difference in paw withdrawal latencies in response to cutaneous thermal stimulation in the surgeried paw to the non-surgeried paw following sham/CCI surgery	%	W = 0.911, p = 0.189	Average withdrawal latency in the surgeried paw expressed as a percentage of withdrawal latency in the non-surgeried paw across the three test sessions (days 63 to 65) following sham/CCI surgery	Values close to 100% reflect relatively similar levels of thermal pain in the two paws. Low values reflect higher pain in the surgeried paw
Reflex pain–Δ surg. paw	Change in paw withdrawal latency in response to cutaneous thermal stimulation in the surgeried paw following sham/CCI surgery	s	W = 0.964, p = 0.813	Subtraction of withdrawal latency in the surgeried paw on day 6 from “Reflex pain–surg. paw”	High negative values reflect larger increases in cutaneous thermal pain sensitivity
CPP–Δ pre-test CPP	CPP following sham/CCI surgery relative to Pre-test	s	W = 0.947, p = 0.554	Subtraction of Pre-test (day 10) time in the OXY-paired compartment from time in the OXY-paired compartment after sham/CCI surgery (day 66)	High values reflect larger CPP
CPP–Δ post-cond. CPP	CPP following sham/CCI surgery relative to CPP expressed immediately following conditioning	s	W = 0.954, p = 0.665	Subtraction of time in the OXY-paired compartment immediately after conditioning (day 17) from time in the OXY-paired compartment after sham/CCI surgery (day 66)	High values reflect larger CPP relative to that immediately following conditioning
CPP–Δ pre-surg. CPP	CPP following sham/CCI surgery relative to CPP expressed immediately before sham/CCI surgery	s	W = 0.936, p = 0.411	Subtraction of time in the OXY-paired compartment at the end of extinction testing (day 51) from time in the OXY-paired compartment after sham/CCI surgery (day 66)	High values reflect larger CPP relative to that expressed at the end of extinction

### Animals and drugs

Fifteen Sprague–Dawley rats (7 male, 8 female) entered the study, which was reduced to 13 (7 male, 6 female) following sham/CCI due to complications with surgery. To reduce the impact of differences in body weight between the sexes, all animals were delivered from the supplier (Envigo, Indianapolis, IN, USA) to the University of Florida vivarium weighing between 200 and 225 g (ages 8 and 12 weeks for males and females respectively). Mean (± standard deviation) animal weight on day 13 of the study was 249.4 ± 38.6 g. Animals were pair-housed by sex, provided with standard laboratory chow and water ad libitum, and maintained on a 12:12-h light/dark cycle. Behavioral testing procedures took place approximately 2 to 8 h into the light phase. All experiments were conducted in accordance with the Guide for the Care and Use of Laboratory Animals and were reviewed and approved by the University of Florida Institutional Animal Care and Use Committee. As males and female animals differed in age (to enable comparable weights at the start of testing), data are presented pooled across the sexes. OXY hydrochloride was prepared by dissolving in phosphate buffered saline vehicle (VEH) at a concentration of 0.56 mg/ml, corrected for salt content and administered intraperitoneally (i.p.).

### Thermal hindpaw withdrawal reflex pain testing procedure

Animals were acclimated to a thermal hindpaw reflex testing apparatus (Ugo Basile model 7375a, Stoelting, Wood Dale, IL, USA) for at least 10 min. A radiant heat lamp (intensity set at 45) was targeted on the left or right hind paw (random order) until the paw was withdrawn, using a cutoff time of 20 s to prevent tissue damage. Two or more paw withdrawal latency measures (minimum 5 s each) were taken for each paw, from which the two numerically closest values were averaged.

### Induction and extinction of CPP

Place conditioning studies were performed in a sex-counterbalanced order in a three-compartment place conditioning apparatus (Med Associates, model ENV-013, Fairfax, VT, USA). The apparatus consisted of two 28 × 21 × 21 cm outer conditioning compartments separated by a 12 × 21 × 21 cm center compartment. One outer compartment had black walls on which large (11 cm) white stars were superimposed. This compartment also had a floor made from 4.8 mm stainless steel rods, placed at 16 mm intervals. The other compartment had white walls on which large black circles (9 cm) were superimposed. This compartment also had a 1.25 × 1.25 cm^2^ stainless steel mesh floor. The center compartment had gray walls and a smooth polyvinyl chloride floor. Animal position and locomotion were recorded using photobeam breaks. Before conditioning, a 20 min initial preference test (termed “Pre-test”, day 10) was performed in which animals could freely explore all three compartments after a 2 min acclimation period in the center compartment, and the amount of time spent in each was recorded. On days 13–16, animals underwent 30 min conditioning sessions in their assigned compartments. OXY was administered in one compartment and VEH was administered in the other. Approximately equal numbers of animals were assigned to each compartment in a pseudorandom counterbalanced fashion based on their Pre-test times, which ensured no statistical difference between the mean Pre-test time of each group between the compartments. Injections were given immediately before placement in the compartments. To minimize order effects, animals were counterbalanced such that during morning conditioning sessions, half received OXY and the other half received VEH, with the opposite respective conditioning treatment occurring approximately 4 h later. Ensuring a fully unbiased conditioning protocol obviated the need of an additional group of animals that received VEH in both conditioning compartments to demonstrate acquisition of OXY-induced CPP. Horizontal locomotor activity in response to VEH and OXY administration was recorded as beam break counts during conditioning sessions. The day following conditioning (day 17), and periodically thereafter (days 24, 28, 38, 45, 49, 51), animals were tested for expression of CPP in the same manner as the Pre-test. On day 66, which occurred after CCI or sham surgery, animals were re-tested a final time to ascertain if a chronic pain state reinstated expression of CPP.

### Chronic constriction injury (CCI) surgery

Chromic gut suture (4.0, Ethicon) was cut into 2 cm pieces and immersed in sterile saline to prevent drying. Surgery was performed under aseptic conditions on a T-pump heating pad. Animals were administered buprenorphine (SR)b (1.0 mg/kg s.c.) before being maintained under general anesthesia (3–5% isoflurane in O_2_). The right hind leg (referred to as the “surgeried paw” vs. the left hind leg which is referred to as the “unsurgeried paw”) was shaved and sterilized with chlorohexidine and 70% ethanol. A 5 to 7 mm incision was made in the skin below the femur and the skin separated from the muscle and connective tissue using blunt forceps. An incision was made through femoris muscles, and the sciatic nerve freed using a glass pipette with a blunt curved tip. Three ligatures were made using a double knot 1 mm apart, tightened until the loop was just snug and the ligatures were unable to slide along the nerve. Silk sutures (5.0, Ethicon) were used to close the muscle layer and skin, and the wound was cleaned with chlorohexidine before application of triple antibiotic ointment. Following surgery, animals were variably housed with cagemates that had undergone the same or other surgery. “Sham” operated animals underwent the same procedure (i.e. same paw, surgical intervention, treatment etc.) with the exception that no ligatures were placed on the nerve.

### Outcome measures

The main outcome measures of interest (presented in italics throughout text) and their method of calculation are detailed in Fig. 1 and Tables 1 and 2. Paw withdrawal measures across both paws were averaged for each session in the thermal hindpaw test on days 6 to 9, as this was before sham/CCI surgery. As responses during thermal hindpaw testing decreased over the four sessions, the first test session was used for the reflex pain withdrawal latency measurement (termed “*Basal reflex pain*”) for each animal. The rate of decrease in withdrawal latencies (termed “*Rate of change in reflex pain*”) was calculated by fitting a linear regression, with the slope being inverted such that a higher positive value indicated greater rates of decrease.

Locomotor responses following VEH administration in the first conditioning session were taken as a measure of basal locomotion (termed “*Basal loco*”). The rate of locomotor habituation (termed “*Rate of loco habituation*”) during conditioning was calculated by fitting a linear regression to the locomotor activity following the four VEH administrations during conditioning on days 13 to 16, with the slope being inverted such that a higher positive value indicated faster rates of habituation. Of all the outcomes measured, only this measure presented a non-normal distribution. Consequently, values were transformed by taking the square root of the initial values to yield values fitting a normal distribution, thereby allowing consistent use of parametric analysis methods across all measures.

Locomotor responses following OXY administration in the first OXY conditioning session were normalized to locomotor responses following VEH administration during the first VEH conditioning session by subtracting locomotor counts to yield a measure of acute OXY-stimulated locomotion (termed “*Acute OXY loco*”). Rates of behavioral sensitization (termed “*Rate of behavioral sensitization*”) were calculated by first normalizing locomotor responses to OXY to same-day locomotion following VEH administration during conditioning sessions by subtracting the latter from former and then by fitting a linear regression curve to yield a slope reflecting the rate of change of locomotion over the four conditioning sessions.

CPP following conditioning was calculated by subtracting time spent in the OXY-paired compartment before conditioning (i.e. at Pre-test, day 10), from times spent in the OXY-paired compartment after conditioning (i.e. days 17, 24, 28, 38, 45, 49 and 51). The strength of OXY-induced CPP immediately following conditioning (day 17) was termed “*CPP at first test*”. Rates of extinction of CPP (termed “*Rate of CPP extinction*”) were estimated by calculating the slope of a fitted linear regression across all post-conditioning CPP sessions (i.e. days 17, 24, 28, 38, 45, 49 and 51) before sham/CCI surgery. The value of the slope was inverted such that a higher positive value indicated faster rates of extinction.

The impact of sham/CCI surgery on paw withdrawal latencies in the thermal hindpaw test was averaged over three tests, which generated three measures detailed in Fig. 1 and Table 1. These were absolute sensitivity of the surgeried paw (termed “*Reflex pain – surg. paw*”), relative sensitivity of the surgeried paw to the unsurgeried paw (termed “*Reflex pain—surg. paw vs. non-surg. paw*”) and change in sensitivity of the surgeried paw relative to the first thermal hindpaw test (day 6) taken before sham/CCI surgery (termed “*Reflex pain–*Δ* surg. paw*”).

The impact of sham/CCI surgery on expression of extinguished OXY-induced CPP was assessed 4- or 5-days following sham/CCI surgery, counterbalanced across surgery treatment groups. The strength of CPP was expressed in three different ways detailed in Fig. 1 and Table 1. These were: relative to Pre-test (termed “*CPP–*Δ* pre-test CPP*”), relative to the CPP expressed immediately following conditioning (i.e. day 17, termed “*CPP–*Δ* post-cond. CPP*”), and, relative to the CPP expressed immediately before sham/CCI surgery (i.e. day 51, termed “*CPP–*Δ* pre-surg. CPP*”).

### Data analysis

Data are expressed as mean ± standard error of the mean unless otherwise noted and analyzed using parametric statistical methods based on normality testing of individual measures using a Shapiro–Wilk normality test (results shown in Tables 1 and 2). Data were analyzed using Statview (version 5.0.1, SAS Institute) and R (version 4.3.0^36^). Details of individual statistical tests and the rationale for their application are detailed where appropriate. Probability (p) values represent two-tailed testing and are presented uncorrected throughout.

## Results

### General effects in measures obtained before sham/CCI surgery

**Figure 2:**
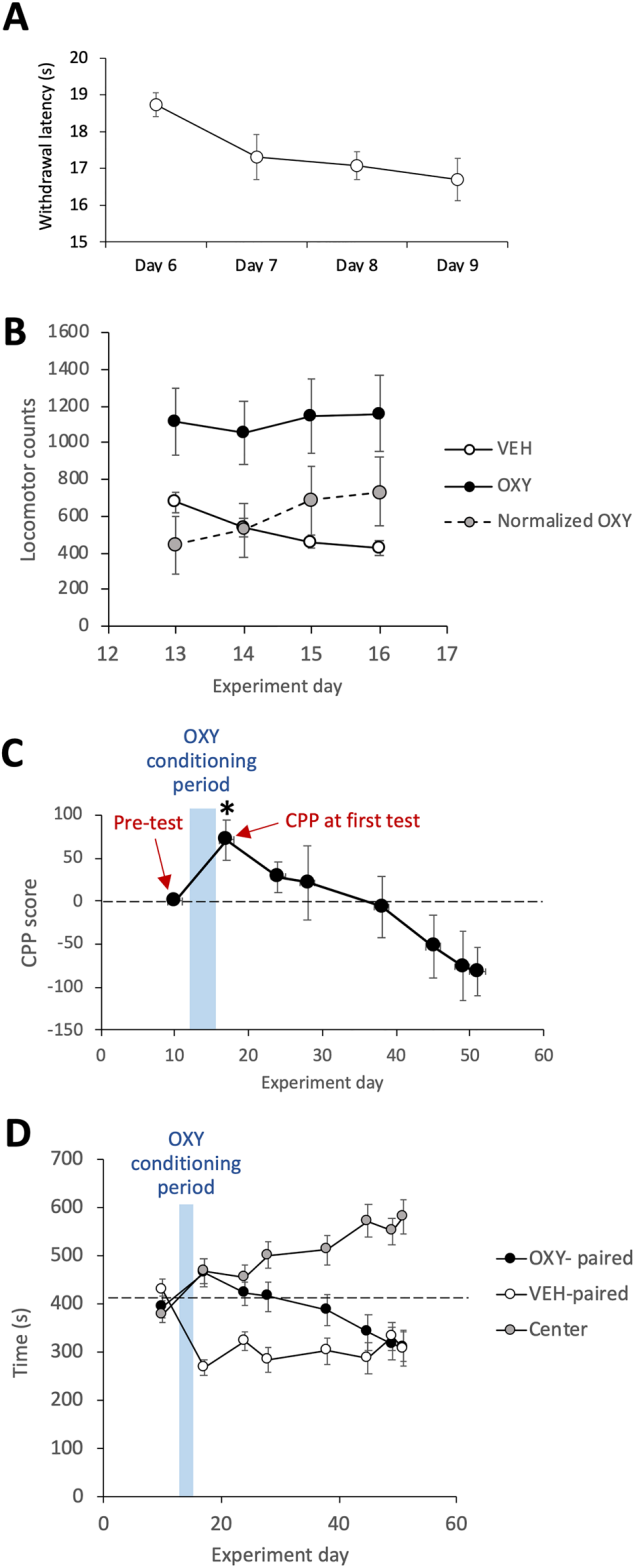
Cutaneous thermal pain sensitivity, locomotion and behavior in the CPP apparatus. (**A**) Changes in paw withdrawal latency (mean of both paws) during repeated cutaneous thermal reflex pain testing. (**B**) Locomotor activity during conditioning in the CPP apparatus. Normalized OXY locomotion was determined by subtracting the number of beam breaks following VEH administration from the number of beam break counts following OXY administration on the same day of conditioning. (**C**) Expression of CPP for the OXY-paired compartment before and after conditioning relative to Pre-test. (**D**) Absolute time spent in the three compartments of the CPP apparatus before and after conditioning illustrating progressively increasing times spent in the center compartment. Data are expressed as mean ± standard error of the mean, n = 15, *p < 0.05 vs. Pre-test.

Repeated measures ANOVA showed paw withdrawal latencies decreased (F_3,42_ = 4.016, p = 0.0134) over the 4 days of testing before induction of CPP from an initial mean of 18.7 ± 0.3 s to a final mean of 16.7 ± 0.6 s (Fig. 2A). Testing the *rate of change in reflex pain* against a hypothesized mean of 0 (i.e. theoretical unchanging withdrawal latencies) yielded a significant difference (t_14_ = 3.451, p = 0.004).

Repeated measures ANOVA showed that locomotion in VEH conditioning sessions progressively and significantly (F_3,42_ = 19.675, p < 0.0001) decreased over the 4 days of testing from a mean of 675.3 ± 56.2 counts to 427.1 ± 39.0 counts, suggestive of locomotor habitation (Fig. 2B). This was further reflected in a mean *rate of locomotor habituation* slope of 8.7 ± 0.7 counts/day (all animals showed positive values), which, when tested against a hypothesized mean of 0, and confirmed a statistically significant (t_14_ = 12.84, p < 0.0001, one sample *t* test) reduction in locomotor activity following VEH administrations across the whole group of animals.

OXY administration on the first day of conditioning induced significantly (t_14_ = 2.882, p = 0.0121, paired *t* test) greater (65.4%) locomotion than VEH administration on the same day (Fig. 2B). Thereafter, 10 of 15 animals showed a positive behavioral sensitization slope (increase in OXY-induced locomotion across days). When considered across all animals, locomotor responses to OXY increased during conditioning sessions (Fig. 2B), yielding a mean *Rate of behavioral sensitization* slope of 104.0 ± 43.5 VEH-normalized counts/session such that normalized OXY-stimulated locomotion increased from 441.7 ± 153.3 counts to 734.0 ± 185.6 counts of locomotion between the first and last conditioning sessions. Testing the *rate of behavioral sensitization* slope against a hypothesized mean of 0 (i.e. theoretical unchanging locomotor activity during repeated OXY administration), yielded a significant difference (t_14_ = 2.39, p = 0.0315, one sample *t* test) indicating behavioral sensitization, though when tested by repeated measures ANOVA over the four administrations of OXY, normalized OXY-stimulated locomotion narrowly missed reaching statistical significance (F_3,42_ = 2.633, p = 0.062).

The average time in the compartment destined for pairing with OXY at pre-test (day 10) exhibited a normal distribution (W_15_ = 0.9668, p = 0.8081) and was 393.4 s (standard deviation, 92.2 s; range 203.1 s to 544.5 s). Groupwise, animals showed no initial bias for either of the two prospective conditioning chambers (t_14_ = 0.808, p = 0.4325, paired *t* test). Three days after the pre-test (days 13 to 16), animals were assigned to receive OXY in one of the two conditioning compartments (seven assigned to one, eight assigned to the other) in a counterbalanced fashion such that no statistical difference in Pre-test times existed between the two assignment groups (t_13_ = 1.537, p = 0.1483, unpaired *t* test). Figure 2C shows CPP scores (respective to Pre-test) measured before and after OXY conditioning. Repeated measures ANOVA applied across all CPP scores, including Pre-test, yielded a significant effect of test session (F_7,98_ = 4.872, p < 0.0001) generally reflecting acquisition and extinction of OXY-induced CPP. A within subjects paired t-test showed a statistically significant (t_14_ = − 3.027, p = 0.0091) increase in time spent in the OXY-paired compartment during *CPP at first test* compared to Pre-test. A within-subjects paired *t* test comparing time spent in the OXY-paired compartment between *CPP at first test* and the final test of CPP before sham/CCI surgery showed a significant (t_14_ = 4.235, p = 0.0008) reduction in CPP, reflecting extinction of CPP. This was further reflected in a mean *Rate of extinction* slope of 4.4 ± 0.9 s/day (14 of 15 animals showed a positive value), which tested against a hypothesized mean of 0 (i.e. theoretical unchanging strength of CPP) confirmed statistically significant (t_14_ = 4.654, p = 0.0004, one sample *t* test) extinction across the whole group of animals. Notably by the final test (day 51) before sham/CCI surgery, animals showed an apparent aversion to the OXY-paired compartment such that CPP scores were significantly lower than zero (t_14_ = 2.953, p = 0.0105, one sample *t* test). Additional analysis showed that this apparent aversion may have been partially driven by progressive increases in the amount of time spent by animals in the center/grey compartment of the CPP apparatus (Fig. 2D).

### Correlational analysis of measures obtained before sham/CCI surgery

**Figure 3:**
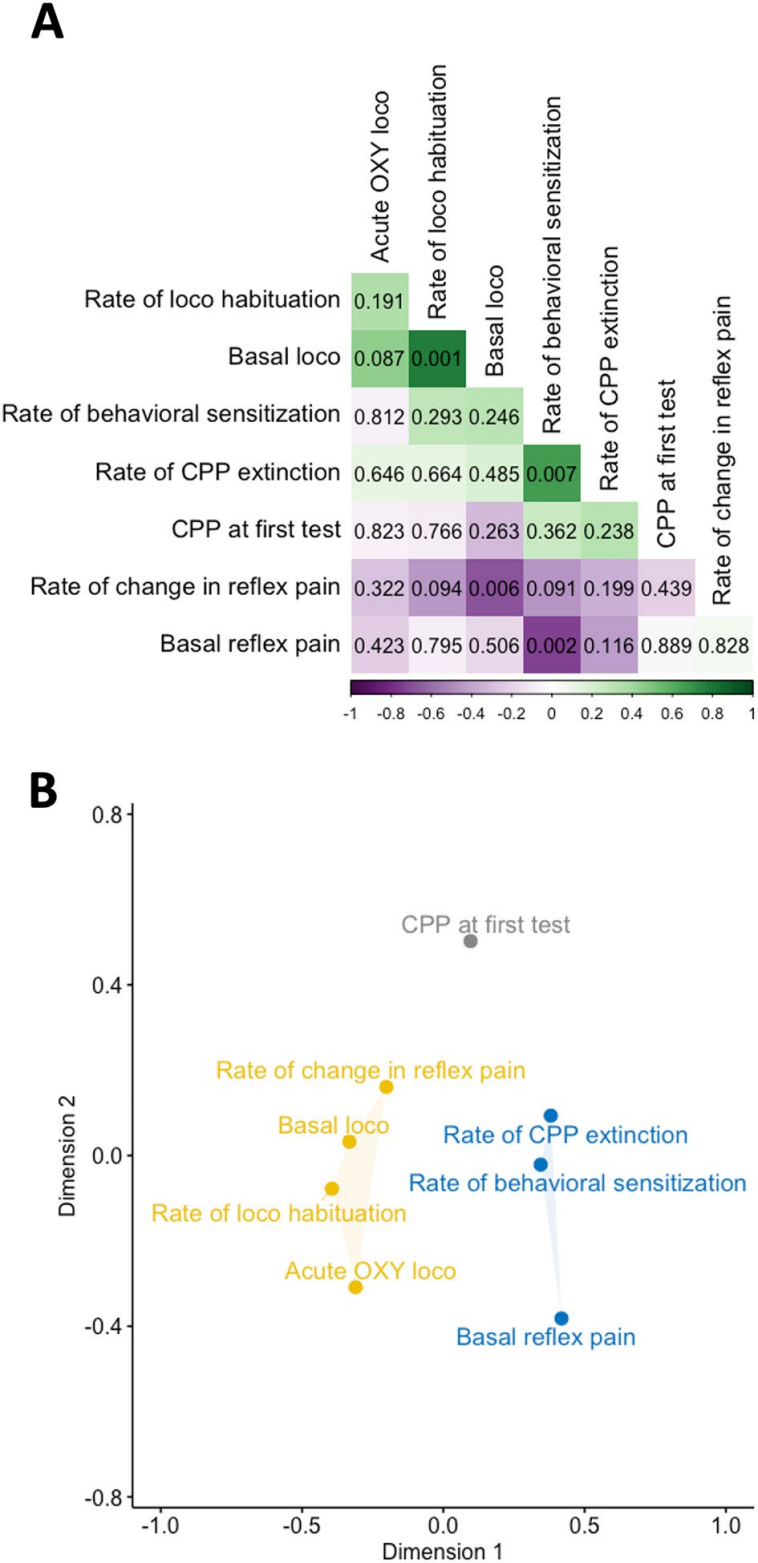
Relationships between behavioral outcomes measured before sham/CCI surgery. (**A**) Correlation matrix of Pearson’s correlation coefficients between major outcomes measured before sham/CCI surgery arranged by angular order of the eigenvectors. Positive correlations are indicated in green. Negative correlations are indicated in purple. More intense coloring indicates higher coefficients, i.e. stronger correlation. Uncorrected p values for each correlation are indicated. (**B**) Classical multidimensional scaling analysis of major outcomes measures. Outcome measures are k-means clustered into three clusters, shown in different colors.

A correlational analysis was performed by generating Pearson's correlation coefficients (using R function *cor*) between all major outcome measures taken prior to sham/CCI surgery. Figure 3A shows a correlation matrix and associated uncorrected p values (calculated using R function *rcorr$P*) arranged by angular order of the eigenvectors such that closely correlated variables are proximate in the matrix. Among all pairwise correlations, four had uncorrected p values lower than 0.05, these being *Basal loco* and *Rate of loco habituation* (positive correlation), *Rate of behavioral sensitization* and *Rate of CPP extinction* (positive correlation), *Rate of change in reflex pain* and *Basal loco* (negative correlation), and, *Basal reflex pain* and *Rate of behavioral sensitization* (negative correlation).

### Data reduction analysis of measures obtained before sham/CCI surgery

To elucidate general relationships between measures regardless of directionality in the relationships, all correlations coefficients (*r*) in the correlation matrix were converted to their absolute values [i.e. abs(*r*)], which was then converted to a distance matrix by subtracting the value from 1 [i.e. 1-abs(*r*)] which was then further subjected to a classical multiple dimensional scaling analysis using the R function *cmdscale*, the results of which are shown in Fig. 3B. Multidimensional scaling reduces data set complexity to visually identify unrecognized dimensions that impact behavior^37^. Here, the pattern of proximities of the major outcome measures based on a distance matrix created from correlational strength is visualized in 2-dimensional abstract Cartesian space. In this space and under these analysis conditions, correlated outcomes, regardless of their positive or negative sign, are represented close together, and uncorrelated outcomes are represented far apart. To objectively assess if inter-outcome relationships could be grouped into those with distinct relationships, the outputs of the multidimensional scaling analysis were subjected to a K-clustering analysis using the R function *kmeans*. K-means clustering applies an unsupervised machine learning algorithm to identify clusters of data objects in a dataset. In this case, clusters of correlated measures are identified. The optimal number of clusters was determined first by applying the output of the multidimensional scaling analysis values to the R package *NbClust*^38^ set to suggest a number of clusters between 2 and 6, which suggested 3 clusters as optimal. As shown in Fig. 3B, this approach clustered *Basal reflex pain, Rate of behavioral sensitization* and *Rate of CPP extinction* into one cluster, *Basal loco*, *Rate of loco habituation, Acute OXY loco* and *Rate of change in reflex pain* into a second cluster, and *CPP at first test* into its own third cluster*.* These results suggest that thermal pain sensitivity relates generally to behavioral sensitization and the persistence of CPP, whereas locomotor responses and rate of change of withdrawal latencies during repeated reflex pain testing are related. The strength of acquired CPP appears to be generally distinct from all other measures.

### Effects of CCI surgery on expression of extinguished OXY-induced CPP

**Figure 4:**
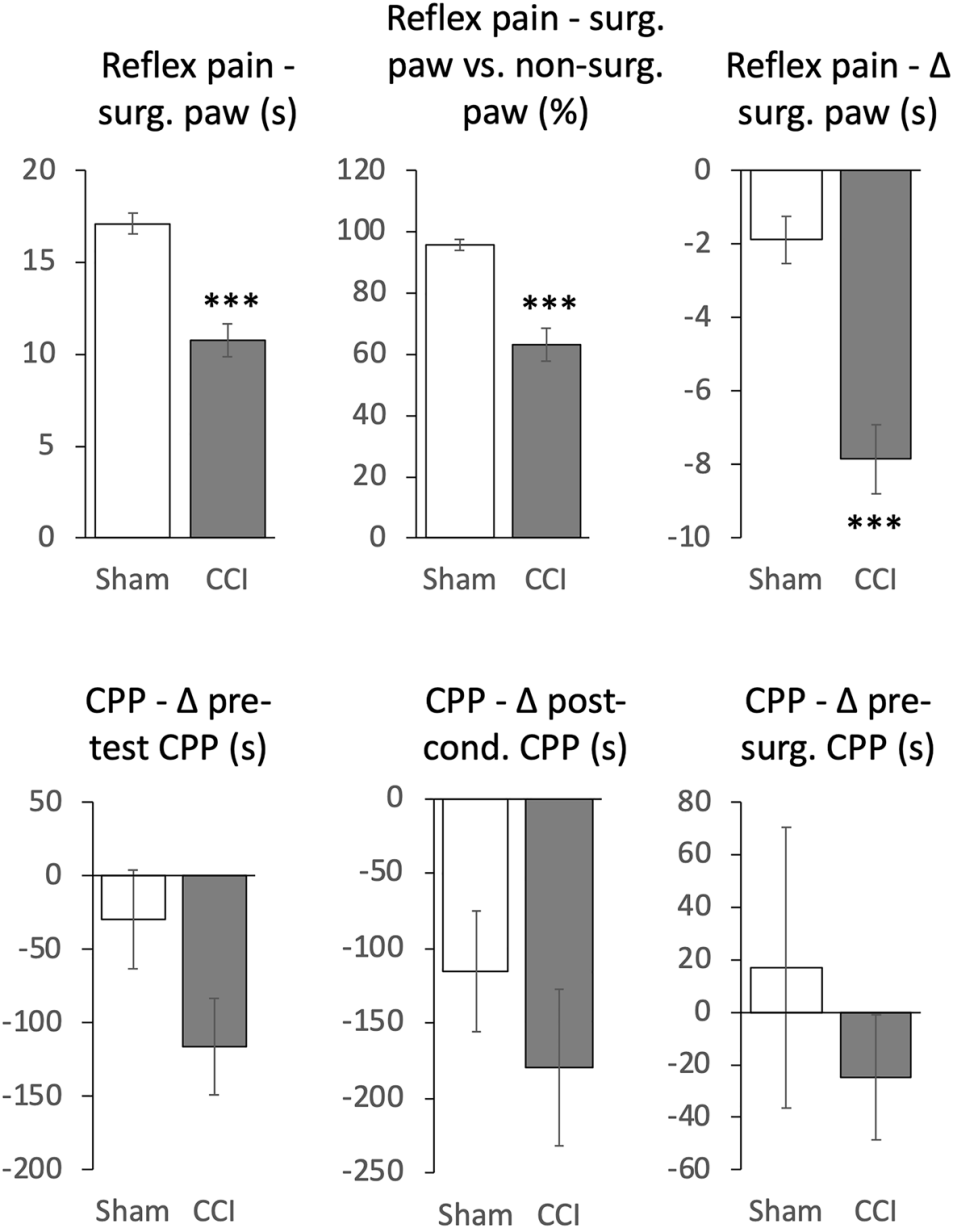
Effect of sham or CCI surgery on outcomes. Data are expressed as mean ± standard error of the mean, n = 6–7/group, ***p < 0.0001 unpaired student’s *t* test except for *CPP–*Δ *post-cond. CPP*, which was compared using a Mann–Whitney *U* test (Z =  − 0.484, p = 0.628)*.* t values for unpaired two-tailed students *t* test comparisons from top left to bottom right respectively, omitting *CPP–*Δ *post-cond. CPP*: t_11_ = 5.866, 5.448, 5.050, 1.837, 0.751.

Except for the *CPP–*Δ* post-cond. CPP* measure in sham treated animals (W = 0.748, p = 0.030), all measures taken after sham/CCI surgery were normally distributed (W = 0.863–0.982, p = 0.176–0.999) when split into treatment groups. Considered across both groups, all measures were normally distributed (W = 0.911–0.964, p = 0.189–0.812, Table 2). Figure 4 shows means for all six measures. CCI-animals showed a strong and statistically significant (unpaired *t* test) reduction in all three reflex pain measures indicating the effectiveness of the surgery to increase nociceptive responding relative to sham animals. By all three CPP measures, CCI animals showed more negative CPPs relative to sham animals, suggesting that the nociceptive state induced by CCI caused animals to avoid the OXY-paired compartment. However, none of the three measures were significantly different (unpaired t-test), though there was a tendency towards significance (t_11_ = 1.837, p = 0.0933) in the *CPP–*Δ* pre-test CPP* measure. Notably for both treatment groups, this measure was negative, which when tested against 0 (one-sample *t* test), yielded a statistically significant avoidance of the OXY-paired compartment in the CCI animals (t_6_ = 3.5459, p = 0.0121) but not in sham animals (t_5_ = 0.8820, p = 0.4182) suggesting CCI surgery produced a stronger aversion to the OXY-paired compartment relative to pre-test than in sham animals.

### Correlational and data reduction analysis of the effects of CCI surgery on expression of extinguished OXY-induced CPP

**Figure 5:**
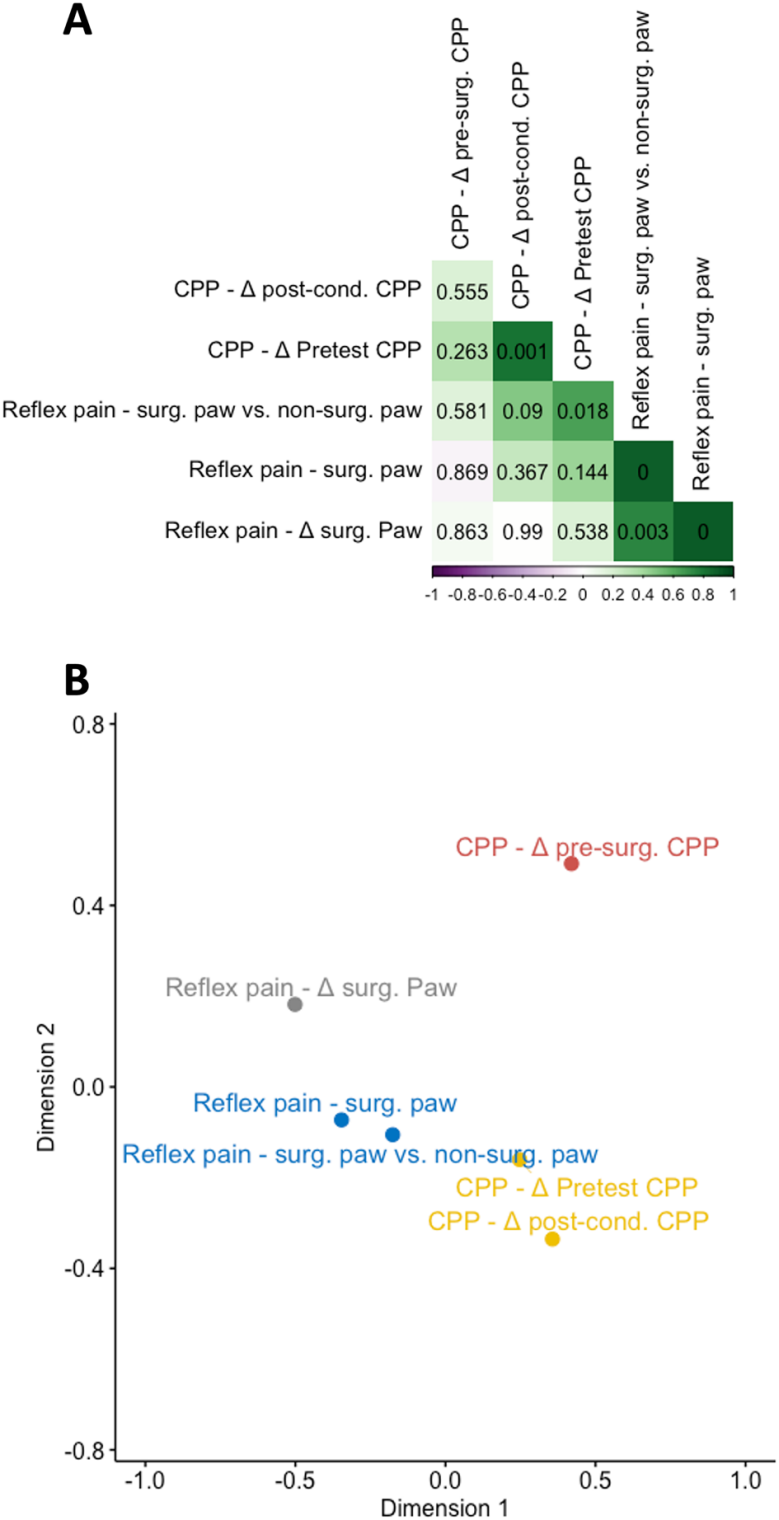
Relationships between behavioral outcomes measured before and after sham/CCI surgery. (**A**) Correlation matrix of Pearson’s correlation coefficients between major outcomes measured after sham/CCI surgery arranged by angular order of the eigenvectors. Positive correlations are indicated in green. Negative correlations are indicated in purple. More intense coloring indicates higher coefficients, i.e. stronger correlation. Uncorrected p values for each correlation are indicated. P values are shown to four decimal places (**B**) Classical multidimensional scaling analysis of major outcomes measures. Outcome measures are k-means clustered into four clusters, shown in different colors.

Figure 5A shows the results of the correlational analysis. Generally, reflex pain measures correlated positively with each other, with some exhibiting p values less than 0.05. Notably, the measures *Reflex pain—surg. paw vs. non-surg. paw* and *CPP–*Δ* pre-test CPP* showed a positive correlation that passed a p threshold of 0.05 (p = 0.018), indicating that those animals with strongest differences in withdrawal latencies between paws in the thermal hindpaw test following sham/CCI surgery showed the strongest avoidance of the OXY-paired compartment relative to pre-test. The R package *NbClust* (set to suggest between 2 and 4 clusters) suggested four clusters as optimal for K-clustering of the multidimensional scaling analysis, the results of which are shown in Fig. 5B.

## Discussion

Here we found that OXY induced CPP, which extinguished during repeated testing. Cutaneous thermal reflex pain poorly predicted OXY-seeking-related behavior in terms of the strength of acquired CPP though related particularly to rates of behavioral sensitization, and chronic pain did not reinstate extinguished CPP. Acute locomotor measures clustered with rates of locomotor habituation and rates of change in reflex pain, and reflex pain clustered with rates of behavioral sensitization and persistence of CPP. Together, these findings suggest that cutaneous thermal pain reactivity is not strongly predictive of OXY reward/seeking as measured by the strength of CPP but may have a relationship with the incentive motivational properties of OXY.

Conditioning with 0.56 mg/kg of OXY produced a significant and persistent CPP response. This is consistent with studies showing that 0.3 mg/kg of OXY is sufficient to generate CPP in rats, though most studies use higher doses^23,39–41^. Notably, the dose used here was chosen for clinical translatability, corresponding to a 6.3 mg dose in a 70 kg human^42^. This falls in the recommended initial prescribing range of 2.5 to 10 mg for chronic pain^43^. Though expression of CPP extinguished throughout repeated testing, our study was not designed to address if this was a passive event or due to repeated re-exposure to the CPP apparatus. Our previous studies in mice suggest the latter, however, in that, left undisturbed, morphine-induced CPP tends to incubate rather than extinguish^44^.

We noted that the locomotor response to initial OXY administration did not correlate with CPP measured immediately following conditioning. This finding is somewhat at odds with the psychomotor stimulant theory of addiction, which hypothesizes that the ability of drugs to stimulate horizontal locomotion predicts their misuse potential^45^. However, others have pointed out that animals often show biphasic dose–response relationships and as such, consideration of dose is critical to the validity of the theory^46^. Relatedly, we found previously that acute OXY administration produces progressive increases in locomotion between 0.178 to 1 mg/kg in the same strain of rat studied here^47^.

An alternative theoretical account designed to explain drug misuse is incentive-sensitization theory^48^. This theory suggests that repeated exposure to misused drugs produces incremental neuroadaptations that render individuals increasingly “sensitized” to drugs and associated stimuli. One of the outcomes of this is progressively increasing locomotor responses, i.e. locomotor behavioral sensitization, with repeated administration. By extension, rates and degrees of behavioral sensitization to a particular stimulus, e.g. drugs, can be predictive of the misuse potential of that stimulus^49^. We previously showed that behavioral sensitization to morphine predicts the strength of CPP in mice^50^. Here, we found only a weak (r = 0.254, p = 0.362) albeit positive correlation between these two outcomes and in the multidimensional scaling analysis the rate of behavioral sensitization did not cluster with strength of OXY-induced CPP. These findings suggest that under these experimental conditions at least, behavioral sensitization to OXY is a poor predictor of the motivating or rewarding properties of OXY.

Drawing together psychomotor stimulant and incentive sensitization theory, we found little direct evidence to suggest a relationship between these two constructs, in that acute locomotor responses to OXY barely correlated with rates of behavioral sensitization and did not appear in the same cluster in the multidimensional scaling analysis. Again, however, species, strain, sex, dose and a range of other factors need to be considered in the interpretation of this negative finding. For instance, previous studies find that acute locomotor responses to cocaine across various rat strains are a poor predictor of behavioral sensitization^51^. Relatedly, there has been considerable interest in the relationship between responses to novelty and drug misuse liability^52–54^. Based on the rationale that initial locomotor responses to VEH injection in the CPP apparatus reflect locomotion driven by novelty, one might expect that this response would predict the strength of expression CPP following OXY conditioning. However, there was in fact a weak negative correlation (r = − 0.309, p = 0.263) between these measures.

A particularly interesting finding of the study is that the rate of extinction of OXY-induced CPP correlated strongly with the rate of behavioral sensitization. Loosely interpreted, this suggests that animals undergoing the most profound adaptations driving drug-seeking behavior more readily experience reversal of those adaptations. It is noteworthy that we found a weak positive correlation (r = 0.324, p = 0.238) between the strength of the initially expressed CPP and rate of extinction. There is the possibility that higher rates of extinction may relate to the stronger initial CPP, providing a greater dynamic range that allows steeper extinction rates. Notably, by the end of extinction testing, animals as a group had developed a significant negative CPP, i.e. apparent avoidance. Given the opportunity to express such avoidance, one could reason that the strength of the initial CPP did not limit the rate of extinction. However, scrutiny of the data in Fig. 2D indicates that the apparent aversion to the OXY-paired compartment was driven largely by animals spending greater proportions of time in the center compartment in which no conditioning had occurred. Some investigators recommend expressing CPP as the proportion of time spent in the drug-paired compartment relative to total time spent in both the VEH and drug-paired compartment^55^. Others calculate CPP scores by subtracting time in the VEH-paired compartment from time in the drug-paired compartment^56^. By this latter method of calculation, CPP at the end of repeated testing was not statistically significantly different from zero (t_14_ = 0.7295, p = 0.4777). This difference draws attention to the impact of differences in calculation methods on the conclusions drawn. As far as we are aware, this issue has not been thoroughly discussed, though others have pointed out theoretical differences between two and three compartment CPP apparatuses^57^.

Though there are many human studies on vulnerability to substance use in pain sufferers, little is known about the relationship between individual differences in pain sensitivity *prior to* developing drug-seeking behavior and substance misuse. An interesting finding of the current study is the evidence of a general relationship between individual sensitivity to thermal reflex pain and some of the behavioral measures acquired during the CPP, in particular a strong negative correlation (r = − 0.728, p = 0.002) between the initial nociceptive response and development of behavioral sensitization. That is, animals most sensitive to cutaneous thermal pain exhibited the most robust behavioral sensitization. This finding agrees with a view that individuals inherently sensitive to pain may be at higher risk of undergoing drug-induced neuroadaptations underlying drug misuse. However, this interpretation should be viewed cautiously, as pain is a multi-dimensional phenomenon, presenting across many modalities, such as nociceptive (of which thermal pain is one), inflammatory, neuroplastic and neuropathic pain^58^. Here, we studied only the relationship between one of these – cutaneous thermal pain – and measures related to drug misuse.

Re-instatement of CPP has been suggested to model some of aspects of relapse, though technically reinstatement of CPP does not model reinstatement of drug-taking in humans as animals do not actually resume drug intake^59,60^. Regardless, various stimuli can reinstate extinguished CPP, notable among these being stress^61^. A particularly common method of demonstrating stress-induced reinstatement is use of foot shock, which is an inherently acute painful stimulus. As far as we are aware, few studies have investigated the effect of chronic pain on reinstatement of CPP, though operant self-administration studies indicate that analgesics can be more reinforcing in animals in chronic pain^62–64^. Here we used an established model of chronic neuropathic pain, CCI, to study the impact of chronic pain on reinstatement of drug-seeking behavior. The impact of CCI on nociceptive responses was clear from its ability to significantly reduce paw withdrawal latencies; however, CCI did not appear to affect reinstatement of extinguished CPP. In fact, CCI animals tended to show *greater* avoidance of the OXY-paired compartment, a finding that concords somewhat with studies showing chronic pain can in some cases reduce the reinforcing and rewarding impact of misused drugs (reviewed in^65,66^) and does not reinstate opioid seeking behavior^67^. Relatedly, nerve ligation suppresses development of morphine CPP in mice^68^. On face value, this finding contradicts a prediction that animals in most pain would seek out OXY to a greater extent. However, it is important to note that animals were not conditioned to OXY in a pain state, and as such are unlikely to associate the OXY-paired compartment with pain relief. Previous studies show that in agreement with the operant self-administration studies introduced above, CPP to morphine is enhanced in animals experiencing a chronic pain state during conditioning^69^. However, studies pairing noxious stimuli with opioid self-administration show that exposure to the noxious stimuli does not reinstate opioid seeking^67^.

There are several caveats requiring consideration in the interpretation of findings in the current study. First, given that the sexes differed in age, data were pooled. However, four of the outcome measures showed statistically different means between the sexes (data not shown). As such, this could cause some correlations to stem from general sex- or age-related differences. Arguing against this, we noted that when split into separate sexes, all correlations with p values less than 0.05 maintained their directionality, suggesting that even though sex/age differences in means exist, the direction of correlations remain. Second, various methods have been used to define acute horizontal locomotor responses, behavioral sensitization, and establishment of CPP. Here we used time in the OXY-paired compartment as our measure of CPP. As introduced above, some studies express CPP as the difference in time spent in the OXY and VEH paired compartment. Our choice of using absolute measures in the OXY-paired compartment to define the existence of CPP was based on the conservative view that ultimately time spent in this compartment alone most simply defines “preference” for the OXY-paired compartment. This method has been applied elsewhere in studies of OXY CPP in mice^21^. Third, outcome measures rarely reflect pure processes. For instance, locomotor responses following VEH and OXY administration could reflect animal handling and injection procedures to some degree. Changes in paw withdrawal latencies during repeated reflex pain testing could partially reflect sensitization to nociceptive pain or habituation to the apparatus and test. Finally, even though animals were derived from different litters as far as possible, genetic variation may not be sufficient to uncover some correlations between behaviors, especially when group sizes are small.

In summary, we studied inter-relationships between behavioral phenotypes relevant to pain sensitivity, drug reward, and drug misuse. We found the strongest evidence of inter-relationships between (1) acute locomotion-related measures and some habituation-like responses, and (2) reflex pain sensitivity, behavioral sensitization and extinction of CPP. Placing animals in a chronic pain state did not re-instate extinguished OXY-seeking behavior. These findings help elucidate relationships between commonly acquired preclinical measures of pain and reward, and suggest that chronic pain does not reinstate drug seeking behavior.

## Data Availability

The dataset used in the current study and R scripts used for analysis and presentation are available from the corresponding author on request.
